# PBAT is biodegradable but what about the toxicity of its biodegradation products?

**DOI:** 10.1007/s00894-024-06066-0

**Published:** 2024-07-18

**Authors:** Ana Martínez, Emiliano Perez-Sanchez, Alexis Caballero, Rodrigo Ramírez, Esperanza Quevedo, Diana Salvador-García

**Affiliations:** Departamento de Materiales de Baja Dimensionalidad, Instituto de Investigaciones en Materiales, Universidad Nacional Autónoma de México, Circuito Exterior S. N. Ciudad Universitaria, 04510 CDMX Mexico City, México

**Keywords:** 1,4-Butanediol, Adipic acid, Terephthalic acid, TPA, TBT, TBTBT

## Abstract

**Context:**

Poly(butylene adipate-co-terephthalate) (PBAT) is a biodegradable plastic. It was introduced to the plastics market in 1998 and since then has been widely used around the world. The main idea of this research is to perform quantum chemical calculations to study the potential toxicity of PBAT and its degradation products. We analyzed the electron transfer capacity to determine its potential toxicity. We found that biodegradable products formed with benzene rings are as good electron acceptors as PBAT and OOH^•^. Our results indicate that the biodegradation products are potentially as toxic as PBAT. This might explain why biodegradation products alter the photosynthetic system of plants and inhibit their growth. From this and other previous investigations, we can think that biodegradable plastics could represent a potential environmental risk.

**Methods:**

All DFT computations were performed using the Gaussian16 at M062x/6–311 + g(2d,p) level of theory without symmetry constraints. Electro-donating (ω-) and electro-accepting (ω +) powers were used as response functions.

## Introduction

Over the years, the petrochemical industry has produced an enormous number of polymers or plastics that undoubtedly contribute to increasing the comfort of humanity. Ten percent of fossil hydrocarbons are transformed into polymers, producing 350 Mt each year [1]. Most are used in packaging, but they are also related to health since they are used to manufacture syringes, condoms, blood bags, catheters, etc. We also use polymers to make coats, avoiding killing animals to protect ourselves from weather conditions. Polymers are everywhere and affect every aspect of our lives, but once used, most of them become garbage [1]. Humanity produces 1.1 Gt per year of waste, of which 10% is plastic [2]. This means that discarded plastics represent a huge and universal pollution problem. One possible solution is to recycle plastic waste up to that become useful feedstock materials. To do this, innovation is required, especially for those plastics for which there is no recycling infrastructure. Today, the cost of producing virgin plastic is much lower than the cost of producing recycled plastic. Resins recovered from plastics through advanced recycling systems are 1.6 times more expensive than virgin resins [3].

Recycling plastics alone is not a global solution, because in addition to the cost, it seems impossible to recover all the plastics that have already been used and discarded. In such a situation, an effective way to reduce plastic pollution is with biodegradable plastics. Biodegradable plastics have become increasingly popular as a potential solution to plastic pollution. The decomposition of biodegradable plastics is due to the action of natural microorganisms such as fungi, algae, and bacteria [4–11]. Among all the biodegradable plastics, there are aliphatic–aromatic co-polyesters, which combines the biodegradability of aliphatic polymers with the good properties of aromatic polyesters. One of the most important of these polymers is PBAT (poly(butylene adipate-co-terephthalate)). PBAT has desirable properties and competitive production costs. It is used in many fields such as food packaging, agricultural, and textile industry [12–18]. It was introduced to the plastics market in 1998 and has been widely used around the world since then [19–21].

The production of PBAT is based in the polycondensation reaction of 1,4-butanediol (BDO), adipic acid (AA), and terephthalic acid (PTA). These reagents come from the petrochemical industry. The degradation process of PBAT involves hydrolysis of the ester bond and seems to be the opposite of this aggregation. Hydrolysis is the first step of degradation and leads to the formation of smaller oligomers that are soluble in water. The second step is microbial degradation [10, 19–22]. PBAT is considered compostable since polyesters are susceptible to enzymatic degradation by esterase [10] but the natural degradation rate of PBAT is slow. As a consequence, it accumulates as waste and causes serious environmental problems [8, 9, 23]. Furthermore, toxicity research has shown that PBAT degradation products may be more toxic than PBAT microplastics [7, 9, 23–29].

Despite all these results, there are no theoretical investigations about the potential toxicity of PBAT and its degradation products. For this reason, the main idea of this research is to perform quantum chemical calculations to study the potential toxicity of PBAT and its degradation products. To do this, we analyze the electron transfer. For polymers, we use oligomers as models and the monomer for PBAT. Oligomers have previously been used to represent polymers with success [30–32]. The results of this investigation can help to understand the possible health effects of biodegradable plastics and to determine which of the degradation products is potentially most dangerous.

## Computational details

Gaussian16 was used for all electronic calculations [33]. Linear structures were considered as initial conformations. Geometry optimizations were obtained at M06-2X/6–311 + g (2d, p) level of theory without symmetry constraints [34–36].

This level of theory has been shown to be adequate for the study of these systems [37, 38]. This exchange–correlation functional was used before with success. This is a global hybrid functional with 54% HF exchange, and it is the best within the 06 functionals for main group thermochemistry, kinetics, and non-covalent interactions. Harmonic analyses verified local minima. In this investigation, the presence of solvent was not considered.

Conceptual density functional theory is a chemical reactivity theory founded on density functional theory-based concepts [39–42]. Within this theory, there are global response functions such as the electro-donating (ω-) and electro-accepting (ω +) powers, previously reported by Gázquez et al. [40, 41]. The capacity to donate electrons (ω-) and the propensity to accept electrons (ω +) are defined as follows:

1 $$\omega -={\left(3I+A\right)}^{2}/16\left(I-A\right)$$

2 $$\omega +={\left(I+3A\right)}^{2}/16\left(I-A\right)$$

I and A are vertical ionization energy and vertical electron affinity, respectively. They are obtained as follows:

3 $$\begin{array}{cc}Y\to {Y}^{+1}+1{e}^{-}& I=E\left({Y}^{+1}\right)-E\left(Y\right)\end{array}$$

4 $$\begin{array}{cc}{Y}^{-1}\to Y+1{e}^{-}& A=E\left(Y\right)-E\left({Y}^{-1}\right)\end{array}$$

**Fig. 1:**
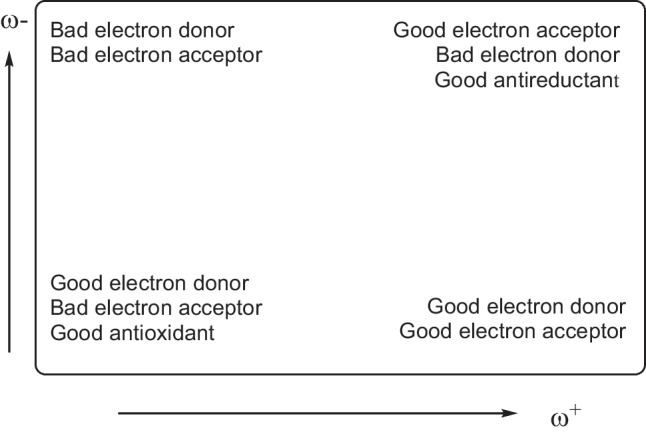
Electron donor–acceptor map (DAM)

Low values of ω- indicate good electron donor molecules. High values of ω + are for good electron acceptor molecules. These two quantities refer to charge transfers, not necessarily from one electron. These chemical descriptors have been used successfully in different chemical systems [43–50]. With these parameters, it is possible to determine the electron donor–acceptor map (DAM, see Fig. 1) [48]. Systems located down to the left are considered good electron donors while those situated up to the right are good electron acceptors.

## Results and discussion

**Fig. 2:**
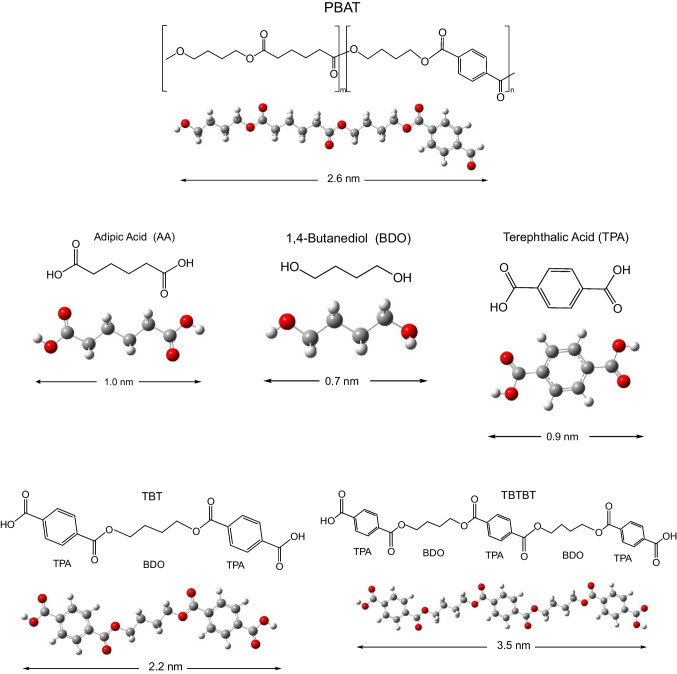
Molecular structures and optimized geometries of PBAT and biodegradation products. Length of the molecules is also reported in nm

Molecular formulas and optimized structures of the studied compounds are shown in Fig. 2. The degradation process of PBAT involves hydrolysis of the ester bond, which is the first step of degradation, and leads to the formation of oligomers. The second step is microbial degradation. Other biodegradation products containing benzene rings (terephthalic acid-butanediol-terephthalic acid (TBT) and terephthalic acid-butanediol-terephthalic acid-butanediol-terephthalic acid (TBTBT) see Fig. 2) were previously detected [22], with TBT being the most abundant. These last two compounds were proposed as biodegradation products to explain the intensity of the fluorescence signals. Authors speculated that the increase in fluorescence intensity was caused by the formation of aggregation oligomers with conjugated soluble benzene rings. The same authors determined two main degradation products with liquid chromatography-mass spectrometry: BDO monomer, the main one that cannot be detected by fluorescence; and the terephthalic acid-butanediol-terephthalic acid (TBT) chain segment that presents fluorescence activity.

Optimized structures presented in Fig. 2 are quasi-planar. The length of the compounds is also indicated and shows that TBTBT is the largest. To obtain information about the toxicity of these compounds, we analyzed the electron transfer capacity. Electron transfer capacity may be related to oxidation of biomolecules. Compounds that accept electrons oxidize other molecules that lose them. Being a good electron acceptor indicates that the molecule is capable of removing electrons from other molecules and therefore oxidize them. Oxidation of biomolecules is related with the transfer of electrons since they occur when there is an excess of free radicals, such as OOH^•^, and free radicals oxidize other biomolecules. This can cause damage to organs and tissues [52–54].

**Fig. 3:**
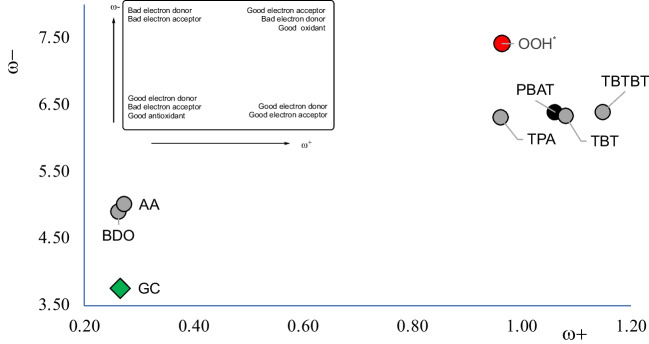
DAM of the products under study. Values are in eV

Figure 3 reports the DAM of the studied products. We also include guanine-cytosine DNA nitrogen base pair (GC) and OOH^•^ for comparison. Table 1 reports the ionization energies (I), electron affinities (A), and the values of the electron donor and acceptor powers (ω- and ω +). All compounds with benzene rings are good electron acceptors. BDO and AA are good electron donors, as GC. The electron acceptor capacity (ω +) of compounds containing benzene rings is similar to the electron acceptor capacity of OOH^•^, that is recognized as a dangerous free radical related to oxidative stress. All good electron acceptor molecules may accept electrons from GC and might induce health problems. It has been previously reported that free radicals were able to damage DNA [52, 53]. Should this be the case, biodegradation products containing benzene rings, which can also steal electrons from the GC, are potentially more toxic than BDO and AA. BDO is the most abundant biodegradation product but apparently, it is not the most toxic when considering the electron transfer process.

**Table 1:** Ionization energies (I), electron affinities (A), and the values of the electron donor and acceptor powers (ω- and ω +). All values in eV

	I	A	ω-	ω +
PBAT	9.81	0.84	6.39	1.06
1,4-Butanediol	10.44	− 1.15	4.91	0.26
Adipic acid	10.64	− 1.15	5.01	0.27
TPA	10.03	0.66	6.31	0.96
TBT	9.62	0.89	6.33	1.08
TBTBT	9.48	1.00	6.39	1.15
GC	7.54	− 0.56	3.76	0.27
OOH	12.51	0.39	7.42	0.96

Comparing products containing benzene rings, TBTBT is the best electron acceptor. According to our arguments, this could be the most dangerous product. PBAT and TBT have a similar electron acceptor capacity, while TPA is a worse electron acceptor and therefore less potentially dangerous than PBAT. Similar results were found with polyethylene terephthalate (PET) [32], which also possesses benzene rings. PET was reported as a better electron acceptor than polyethylene with values similar to those for OOH^•^. Therefore, PET was reported as potentially more toxic than polyethylene. Aromatic polyesters have good properties that allow us to produce very useful plastics, but they could be more harmful than polymers without aromatic molecules because they are better electron acceptors. This is something that should be considered in further analysis of potential toxicity and in future designs of new plastics.

Analyzing all the compounds in Fig. 3, GC is the best electron donor. BDO and AA are worse electron donors than GC. All good electron donors have a very similar electron-accepting capacity (similar values of ω +). The electron transfer from BDO and AA to GC is highly unlikely, which can be interpreted as a lower toxicity of BDO and AA towards the DNA nitrogen base pair. Moreover, the electron acceptor capacity of BDO and AA is not similar to the electron acceptor capacity of OOH^•^ so they cannot be considered as oxidants. In summary, electron transfer capacity indicates that PBAT has similar toxicity as the biodegradation products that have benzene rings. PBAT, TPA, TBT, and TBTBT are the best electron acceptors of all the studied compounds, and they may produce oxidation of biomolecules. BDO and AA are apparently less dangerous considering the electron acceptor capacity.

It was previously reported [18, 24, 25] that BDO, AA, and TPA alter the photosynthetic system of plants and inhibit their growth. They also increase oxidative stress. This is in agreement with our results for TPA, which is a good electron acceptor and therefore could increase the oxidation. BDO and AA are good electron donors and may not participate in oxidation processes. In those previous experiments, the authors did not consider the formation of TBT and TBTBT.

According to the fluorescence results [22], TBT is formed after 5 days under experimental conditions. In 15 days of the experiment, TBTBT is also formed. In another toxicity testing experiment, the authors used plants and exposed them to PBAT for 35 days [25]. The authors of this publication found an inhibition of plant growth and pointed to PBAT as responsible for this. They did not consider the formation of biodegradation products, which were previously reported (TBT and TBTBT). Even when the experimental conditions were not the same in both experiments, the formation of biodegradation products during the plant experiments cannot be ruled out. Growth inhibition could occur through PBAT and by its biodegradation products. If this were the case, the biodegradation products would be as dangerous as PBAT. More experiments are needed to confirm this hypothesis, but our results indicate that the biodegradation products are at least as toxic as PBAT.

## Conclusion

The potential toxicity of PBAT and its biodegradable products can be analyzed through the electron transfer capacity. PBAT, TPA, TBT, and TBTBT are good electron acceptors. BDO and AA are good electron donors. Good electron acceptors stole electrons from other molecules oxidizing them, and they could be more toxic than good electron donors because they may oxidize biomolecules. BDO and AA are apparently less dangerous considering the electron acceptor capacity, but they can dissociate GC.

All these results are in agreement with previous toxicity research which conclude that PBAT degradation products may be more toxic than PBAT microplastics [7, 9, 23–29]. More studies are necessary to analyze other reaction mechanisms to explain the toxicity, but with the theoretical results included here the conclusion is that these compounds can be dangerous because of the oxidation of biomolecules they can produce (electron acceptor molecules).

It was previously reported that BDO, AA, and TPA alter the photosynthetic system of plants and inhibit their growth. They also increase oxidative stress. This is in agreement with our results for TPA, which is a good electron acceptor and therefore could increase oxidation. BDO and AA are good electron donors and may not participate in oxidative stress. In those previous experiments, the authors did not consider the formation of TBT and TBTBT. According to the results reported here, TBT and TBTBT could also increase the oxidation of biomolecules, and they may produce an effect inhibiting the growth of plants.

Our findings show that PBAT may be degraded to produce chemicals that are toxic. PBAT and its degradation products present similar toxicity concerning oxidative stress. Biodegradable plastics may pose a great risk to the environment since the products of degradation might be also toxic. Further studies are necessary to complete the biochemical study using computational chemistry. These results give us insight into possible interactions that should be important for future work.

## Data Availability

Under request.
